# Overexpression of PKM2 promotes mitochondrial fusion through attenuated p53 stability

**DOI:** 10.18632/oncotarget.12942

**Published:** 2016-10-27

**Authors:** Haili Wu, Peng Yang, Wanglai Hu, Yingying Wang, Yangxu Lu, Lichao Zhang, Yongsheng Fan, Hong Xiao, Zhuoyu Li

**Affiliations:** ^1^ Institute of Biotechnology, Key Laboratory of Chemical Biology and Molecular Engineering of National Ministry of Education, Shanxi University, Taiyuan 030006, China; ^2^ College of Life Science, Zhejiang Chinese Medical University, Hangzhou 310053, China; ^3^ Department of Immunology, School of Basic Medicine, Anhui Medical University, Hefei 230032, China; ^4^ College of Life Science, Shanxi University, Taiyuan 030006, China; ^5^ The first hospital of Shanxi Medical University, Taiyuan 030006, China

**Keywords:** PKM2, Drp1, mitochondrial fusion, p53 stability

## Abstract

M2-type pyruvate kinase (PKM2) contributes to the Warburg effect. However, it remains unknown as to whether PKM2 has an inhibitory effect on mitochondrial function. We report in this work that PKM2 overexpression inhibits the expression of Drp1 and results in the mitochondrial fusion. The ATP production was found to be decreased, the mtDNA copy number elevated and the expression level of electron transport chain (ETC) complex I, III, V depressed in PKM2 overexpressed cells. PKM2 overexpression showed a decreased p53 protein level and a shorter p53 half-life. In contrast, PKM2 knockdown resulted in increased p53 expression and prolonged half-life of p53. PKM2 could directly bind with both p53 and MDM2 and promote MDM2-mediated p53 ubiquitination. The dimeric PKM2 significantly suppressed p53 expression compared with the other PKM2 mutants. The reverse relationship between PKM2 and Drp1 was further confirmed in a large number of clinical samples. Taken together, the present results highlight a new mechanism that link PKM2 to mitochondrial function, based on p53-Drp1 axis down regulation, revealing a novel therapeutic target in patients with abnormal mitochondria.

## INTRODUCTION

Altered metabolism is one of the hallmarks that cancer cells differ from normal cells [1, 2]. Unlike normal cells, tumor cells favor high rates of aerobic glycolysis, or referred as the Warburg effect, which generally involves an increased uptake of glucose and the conversion of pyruvate into lactate in the presence of sufficient oxygen [3]. Recent studies indicate that M2 isoform of pyruvate kinase (PKM2), which dephosphorylates phosphoenolpyruvate (PEP) into pyruvate, the last step of glycolysis, plays an important role in determining the cytosolic level of pyruvate, thereby affecting subsequent metabolic flow to lactate, tricarboxylic acid cycle or biosynthetic pathway [4]. Unlike its alternative splicing type PKM1, which is preferentially expressed in adult tissue, PKM2 is frequently highly expressed in embryonic tissues and tumors [5–7]. Although only 22 amino acids are different between PKM1 and PKM2, they are engaged in completely distinct regulatory properties. PKM1 forms a stable, constitutively active tetramer with high pyruvate kinase activity, while PKM2 is dynamically switched between dimer (low pyruvate kinase activity) and tetramer (high pyruvate kinase activity) in tumor cells [8]. Its dimeric form can translocate to nucleus and bind to some transcription factors, subsequently promoting the expression of their downstream genes, which are involved in cancer metabolism and other aspects of tumorigenesis.

Mitochondria are known to play a central role in generating ATP by oxidative phosphorylation (OXPHOS). Warburg suggested that tumor originated from cells with persistent defects in the mitochondrial respiratory system [9]. In the last decades, several groups have investigated the role of mitochondria in the onset of Warburg effect, whereas the correlations between aerobic glycolysis and mitochondrial defection were still not well defined.

Mitochondria are dynamic organelles, and their morphology is governed by the delicate balance between frequent fusion and fission events, and in which multiple proteins mediate the remodeling of the outer and inner mitochondrial membranes [10, 11]. Mitochondrial dynamics are essential for maintenance of mitochondrial morphology and normal function [12]. Deficiency in either fission or fusion reduces mitochondrial trafficking, leading to an aberrant distribution of mitochondria and defective cellular functions. For example, unbalanced fusion leads to mitochondrial elongation, and unbalanced fission leads to excessive mitochondrial fragmentation, both of which impair mitochondrial function [13–15]. Dynamin-related protein-1 (Drp1) is one of the mitochondrial fission proteins and plays a significant role in maintenance of mitochondrial shape, size, distribution and function in mammalian cells. Drp1 can be regulated by post-translational modifications, such as phosphorylation, s-nitrosylation and ubiquitination [16]. In addition, Drp1 can be transcriptionally activated by p53 [17].

The present data demonstrated that elevated PKM2 resulted in defective mitochondria functions by targeting the mitochondrial fission protein Drp1. Mechanically, PKM2 suppressed p53 expression in a proteasome-dependent manner and subsequently its downstream target Drp1 mediated mitochondria fission. Further, the results in patients’ clinical samples are consistent with that *in vitro*. Overall, these data built a new bridge between PKM2 and mitochondrial defection, highlighting the potential of PKM2 as a therapeutic target in tumor.

## RESULTS

### Overexpression of PKM2 triggers mitochondrial fusion

**Figure 1:**
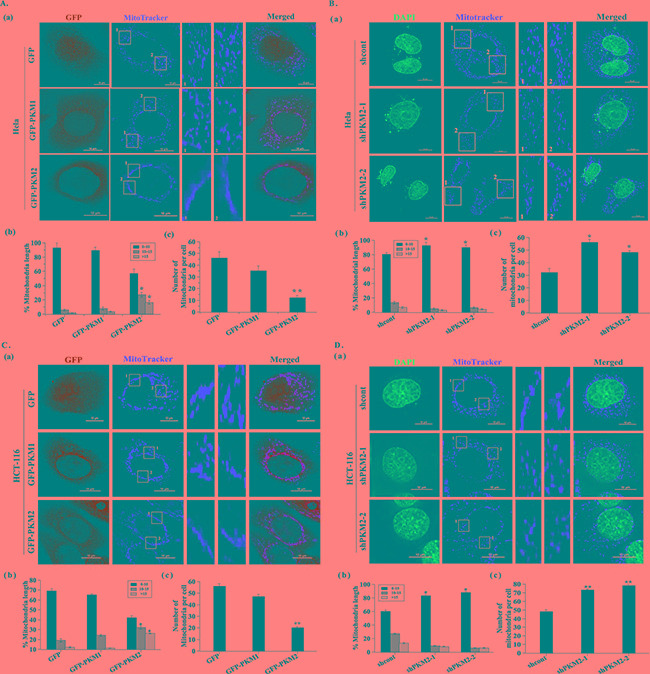
PKM2 induces abnormal mitochondrial morphology **A.** and **C.** (a) Representative images of mitochondrial morphology. HeLa and HCT-116 cells were infected with lentivirus expressing GFP, PKM1or PKM2 for 24 h. Mitochondrial morphology was observed by confocal fluorescent microscope with Mitotracker dye (Green: GFP, Red: Mitotracker dye). Insets are magnifications of the mitochondria at the indicated areas. Scale bar =10 μm. (b) Statistical results of mitochondrial morphology. After transfection 24 h, over 100 transfected cells were categorized into one of three groups depending on mitochondrial morphology. *p < 0.05. (c) Quantitative analysis of mitochondrial number. *p < 0.05, **p < 0.01. **B.** and **D.** (a) Representative images of mitochondrial morphology. HeLa and HCT-116 cells were infected with lentivirus expressing shcont, shPKM2-1or shPKM2-2 for 24 h. Mitochondrial morphology was observed by confocal fluorescent microscope with Mitotracker dye. Cell nuclei were counter-stained by DAPI. Insets are magnifications of the mitochondria at the indicated areas. Scale bar =10 μm. (b) Statistical results of mitochondrial morphology. After transfection 24 h, over 100 cells were categorized into one of three groups depending on mitochondrial morphology. *p < 0.05. (c) Quantitative analysis of mitochondrial number. *p < 0.05.

To determine the relationship of PKM2 and mitochondria, we transfected HeLa and HCT-116 cells with various expression constructs of PKM1, PKM2 or GFP, which was used as the control, and the expression level of PKM1 and PKM2 was detected by western blot (Supplement Figure S1A). The cells were further stained using Mitotracker for observation of the mitochondrial morphology. The results showed that the mitochondria in the GFP and PKM1 overexpressed cells were evenly distributed throughout the cytoplasm. Strikingly, PKM2 overexpressed cells demonstrated a fusion network with elongated mitochondria and an abnormal distribution of mitochondria with most mitochondria clustering around the perinuclear area (Figure 1A (a) and Figure 1C (a)). Statistically, the overexpression of PKM2 showed an increase of elongated mitochondria (Figure 1A (b) and Figure 1C (b)) and a decrease of mitochondrial number (Figure 1A (c) and Figure 1C (c)) as compared to the GFP group. By contrast, the PKM1 overexpressed group did not cause such significant variations on the mitochondrial morphology, length or number. In addtion, the similar mitochondrial changes occurred in another cell line HL-7702 (Supplementary Figure S1C-S1F). In contrast, PKM2 knockdown resulted in more shortened mitochondria and increased mitochondrial density compared to the shcont cells (Figure 1B and 1D). These data demonstrated PKM2 overexpression induced mitochondrial fusion.

### PKM2 induces abnormal mitochondrial fission / fusion events and mitochondrial dysfunction

**Figure 2:**
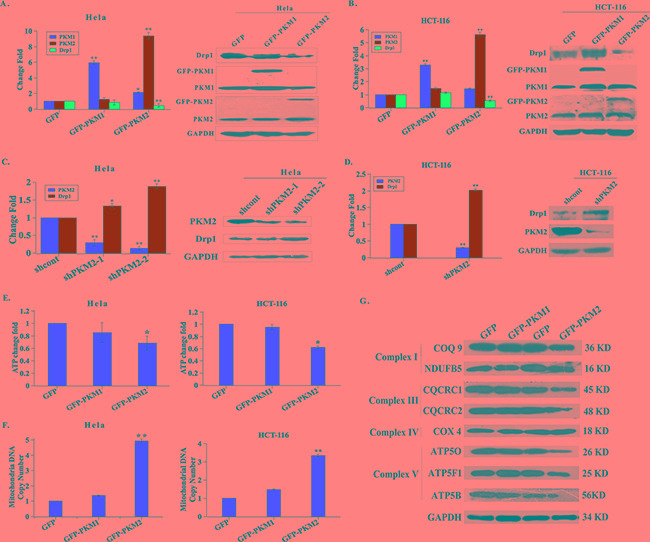
PKM2 induces abnormal mitochondrial fission / fusion events and mitochondrial dysfunction **A.** and **B.** The mRNA and protein expression levels of Drp1 are decreased in PKM2 overexpressed cells. HeLa and HCT-116 cells were infected with GFP, PKM1 or PKM2 lentivirus for 24 h and evaluated by qPCR and western blot. *p < 0.05, **p < 0.01. **C.** and **D.** The mRNA and protein expression levels of Drp1 are increased in PKM2 knockdown cells. HeLa and HCT-116 cells were infected with shcont, shPKM2-1or shPKM2-2 lentivirus for 24 h and evaluated by qPCR and western blot. *p < 0.05, **p < 0.01. **E.** PKM2 overexpression decreases the ATP production. Intracellular ATP was determined using a luciferase-based luminescence assay kit according to the manufacturer's instructions. *p < 0.05, **p < 0.01. **F.** Mitochondrial DNA copy number is increased in PKM2 overexpressed cells. Total DNA was extracted, and relative levels of mtDNA copy number (ND1) were determined by qPCR. The nuclear gene HGB was used for normalization. **p < 0.01. **G.** The expression of ETC complexes is decreased in PKM2 overexpressed cells. HeLa cells were infected with GFP, PKM1 or PKM2 lentivirus for 24 h, then cells were lysed and detected by western blot using anti-COQ9, -NDUFB5, -CQCRC1, -CQCRC2, -COX4, -ATP5O, -ATP5F1 and -ATP5B antibody, respectively. GAPDH was served as a protein loading control.

Given that mitochondrial fusion and fission proteins regulate the mitochondrial morphology and distribution [18], Drp1 induces mitochondrial fission and always functions with other fission/fusion proteins directly or indirectly [19]. We therefore tested whether PKM2-induced mitochondrial elongation was caused through the impairments in the balance of mitochondrial fusion and fission. Subsequently, the expression of mitochondrial fission proteins, Drp1 and Fis, was investigated in PKM2 overexpressed HeLa and HCT-116 cell lines by qPCR analysis. As shown in Figure 2A and 2B, compared to GFP cells, Drp1 expression level was significantly reduced by approximately 0.4- and 0.5-fold in PKM2 overexpressed HeLa and HCT-116 cells, respectively, but no significant changes in PKM1 overexpressed cells were observed. There was no difference with Fis1 expression in both cell lines (Data not shown). Consistent with altered mRNA expression, Drp1 protein level was significantly decreased in PKM2 overexpressed cells, compared to GFP and PKM1 overexpressed cells (Figure 2A and 2B). On the contrary, the mRNA and protein expression levels of Drp1 were largely increased in PKM2-knockdown HeLa and HCT-116 cells (Figure 2C and 2D). At the same time, the similar results were obtained in HL-7702 cells, as shown in Supplementary Figure S2A and S2B. These results suggested that Drp1 was involved in the PKM2-induced abnormal mitochondrial morphology.

Next, we characterized mitochondrial function by assessing mitochondrial ATP production, mtDNA copy number and the expression level of electron transport chain (ETC) complex in PKM2 overexpressed cells. Remarkably, we found that ATP levels were reduced by 35% and 42% in PKM2 overexpressed cells compared to GFP and PKM1 overexpressed HeLa and HCT-116 cells, respectively (Figure 2E). And PKM2-elevated HeLa and HCT-116 cells contained 4.9 and 3.4 fold more copies of mtDNA per nuclear genome than that in the control groups, respectively (Figure 2F). The decreased ATP generation and increased mtDNA copy number were also observed in HL-7702 cells (Supplementary Figure S2C and S2D). The expression level of ETC complex I, III and V was significantly decreased in PKM2 overexpressed cells (Figure 2G). Thus, our data indicated that PKM2 could cause severe mitochondrial dysfunction through diminished expression of Drp1.

### Drp1 was involved in PKM2-induced mitochondrial fusion

**Figure 3:**
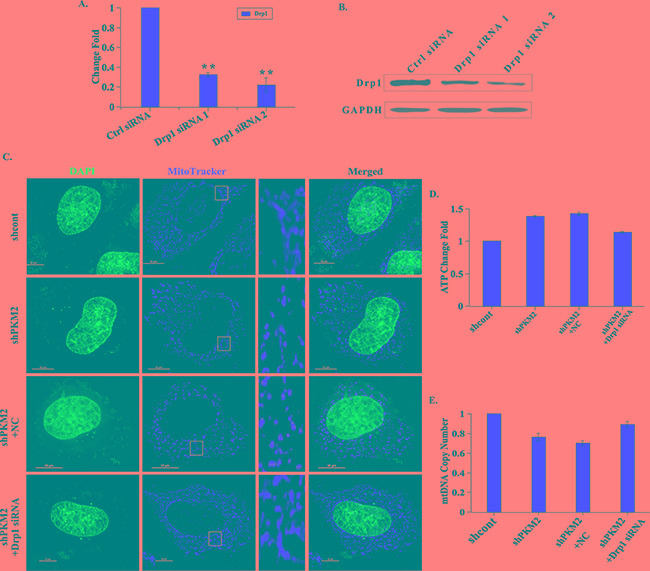
Drp1 is involved in PKM2-induced mitochondrial fusion **A.** and **B.** Drp1 expression is decreased following Drp1 siRNAs treatment. The shPKM2 cells were transfected with Drp1 siRNAs, then cells were harvested, the mRNA and protein expression levels of Drp1 were analyzed by qPCR and western blot, respectively. **p < 0.01. **C.** Drp1 siRNA treatment cells show restored mitochondrial morphology. After the shPKM2 cells were treated with Drp1 siRNAs, mitochondria were stained with Mitotracker dye and the morphology was observed by confocal fluorescent microscope. Insets are magnifications of the mitochondria at the indicated areas. **D.** The ATP production is decreased in Drp1 siRNA treatment cells. After the shPKM2 cells were treated with Drp1 siRNAs, the ATP production was detected. E. The mtDNA copy number is increased in Drp1 siRNA treatment cells. After the shPKM2 cells were treated with Drp1 siRNAs, the mtDNA copy number was detected.

To further determine the involvement of Drp1 in PKM2-induced mitochondrial fusion, we reduced Drp1 expression level by transfecting shPKM2 cells with two different siRNAs against Drp1. Both the qPCR and western blot assay demonstrated that the siRNAs could effectively reduce the expression of Drp1 (Figure 3A and 3B). Notably, the shortened mitochondria in PKM2 knockdown cells were reversed by transfecting with Drp1 siRNAs (Figure 3C). In addition, the ATP production was diminished and mtDNA copy number was elevated following Drp1 siRNA treatment in PKM2 knockdown cells (Figure 3D and 3E). These data indicated that Drp1 was involved in the PKM2-induced mitochondrial dysfunction.

### Overexpression of PKM2 promotes p53 degradation

**Figure 4:**
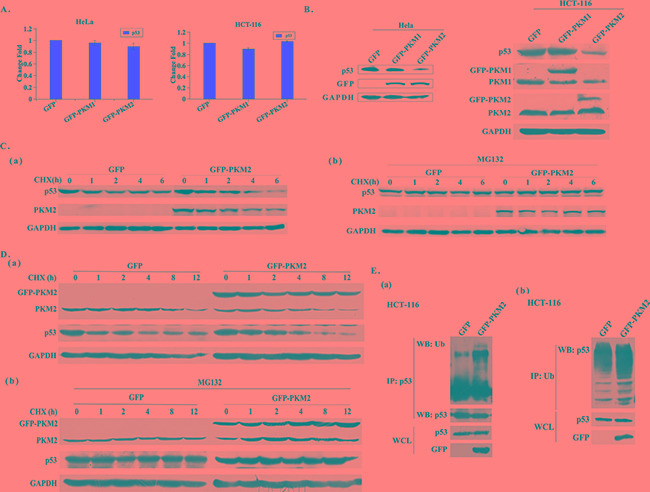
Overexpression of PKM2 promotes p53 degradation **A.** qPCR evaluation of p53 mRNA expression in PKM2 overexpressed HeLa or HCT-116 cells. **B.** Overexpression of PKM2 decreases the level of p53 protein. HeLa or HCT-116 cells were infected with GFP, PKM1 or PKM2 lentivirus for 48 h and cell lysates were analyzed by western blot. **C.** (a) Cells overexpressing PKM2 exhibit a decreased p53 half-life. HeLa cells infected with GFP or PKM2 lentivirus were exposed to cycloheximide (CHX) at a concentration of 20 μg/mL and harvested at the indicated time. Cell lysates were then blotted with p53 antibody. (b) MG132 rescues the decreased level of p53 protein induced by PKM2 overexpression. HeLa cells were infected with GFP or PKM2 lentivirus. After 24 h infection, cells were treated with MG132 (20 μM) for 6 h and CHX (20 μg/mL) for the indicated periods of time, cell lysates were analyzed by western blot. **D.** (a) Cells overexpressing PKM2 exhibit a decreased p53 half-life. HCT-116 cells infected with GFP or PKM2 lentivirus were exposed to cycloheximide (CHX) at a concentration of 20 μg/mL and harvested at the indicated time. Cell lysates were then blotted with p53 antibody. (b) MG132 rescues the decreased level of p53 protein induced by PKM2 overexpression. HCT-116 cells were infected with GFP or PKM2 lentivirus. After 24 h infection, cells were treated with MG132 (20 μM) for 6 h and CHX (20 μg/mL) for the indicated periods of time, cell lysates were analyzed by western blot. **E.** (a) Cells overexpressing PKM2 show increased p53 ubiquitination. HCT-116 cells were infected with GFP or GFP-PKM2 for 24 h and then treated with MG132 (20 μM) for 6 h. Cell lysates were prepared in SDS lysis buffer and p53 proteins were immunoprecipitated to detect ubiquitination. (b) Cells overexpressing PKM2 show increased p53 ubiquitination. HCT-116 cells were infected with GFP or GFP-PKM2 for 24 h and then treated with MG132 (20 μM) for 6 h. Cell lysates were prepared in SDS lysis buffer and ubiquitin proteins were immunoprecipitated to detect ubiquitination.

To explore the mechanism of PKM2 regulating Drp1, we treated the PKM2 overexpressed HeLa cells with the RNA synthesis inhibitor actinomycin D for the indicated times. The stability of Drp1 transcript was not affected by PKM2, suggesting that PKM2 modulated Drp1 mRNA at the transcriptional level (Supplementary Figure S3). In fact, p53 has been well documented to be able to transcriptionally activate the Drp1 by binding to its promoter [17]. To test whether PKM2 affected Drp1 expression through p53, the expression of p53 was detected in PKM2 overexpressed cells. The result showed that no significant change occurred in the transcription level of p53 mRNA (Figure 4A and Supplementary Figure S4A). However, western blot analysis demonstrated that p53 protein level was substantially decreased in PKM2 overexpressed HeLa, HCT-116 and HL-7702 cells (Figure 4B and Supplementary Figure S4A). Based on these findings, we speculated that a post-transcriptional mechanism, such as changes in protein stability, might be involved. Then we evaluated the half-life of p53 protein by time-course western blot analysis in PKM2 overexpressed cells and the control cells after treatment with cycloheximide (CHX, a protein synthesis inhibitor). The result implicated that the half-life of p53 was shorter in PKM2 overexpressed cells (Figure 4C (a) and 4D (a)). We thereby treated PKM2 cells with the proteasome inhibitor MG132. The result showed that the inhibition of proteasomal degradation canceled the difference between the p53 expression of control and PKM2 overexpressed cells (Figure 4C (b) and 4D (b)). Next we examined the ubiquitination of p53 in PKM2-overexpressing cells, and the results indicated that p53 ubiquitination was increased (Figure 4E). All together, these data indicated that PKM2 was important for p53 stability.

### Knockdown of PKM2 induces p53 up-regulation through increased p53 protein stability

**Figure 5:**
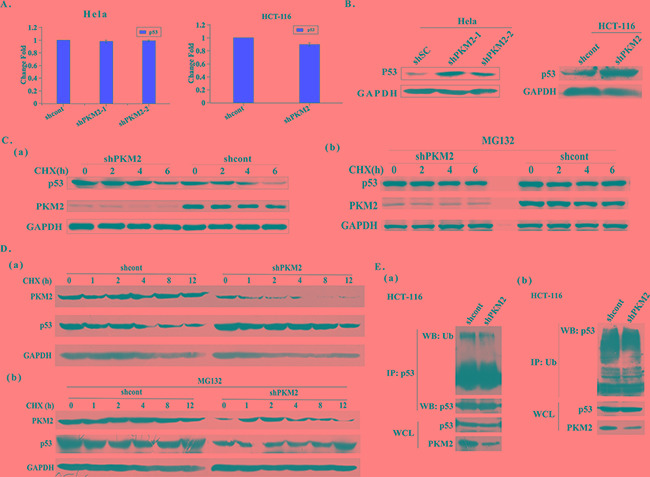
Knockdown of PKM2 induces p53 up-regulation through increasing p53 protein stability **A.** qPCR evaluation of p53 mRNA expression in PKM2 knockdown HeLa or HCT-116 cells. **B.** PKM2 knockdown cells exhibit increased p53 protein levels. HeLa or HCT-116 cells were infected with shcont or shPKM2 lentivirus and harvested after 24 h. Cell lysates were analyzed by western blot. **C.** (a) Knockdown of PKM2 increases the half-life of p53. HeLa cells were infected with shcont or shPKM2 lentivirus for 24 h and then treated with CHX (20 μg/mL). Cells were harvested at the indicated time and lysates were used to detect p53. (b) MG132 rescues the decreased level of p53 protein. HeLa cells were infected with shcont or shPKM2 lentivirus for 24 h, then the cells were treated with MG132 (20 μM) for 6 h and CHX (20 μg/mL) for the indicated periods of time, cell lysates were analyzed by western blot. **D.** (a) Knockdown of PKM2 increases the half-life of p53. HCT-116 cells were infected with shcont or shPKM2 lentivirus for 24 h and then treated with CHX (20 μg/mL). Cells were harvested at the indicated time and lysates were used to detect p53. (b) MG132 rescues the decreased level of p53 protein. HCT-116 cells were infected with shcont or shPKM2 lentivirus for 24 h, then the cells were treated with MG132 (20 μM) for 6 h and CHX (20 μg/mL) for the indicated periods of time, cell lysates were analyzed by western blot. **E.** (a) Knockdown of PKM2 decreases p53 ubiquitination. HCT-116 cells were infected with shcont or shPKM2 lentivirus for 24 h, and then treated with MG132 (20 μM) for 6 h. Cells lysates were prepared by using SDS lysis buffer and p53 proteins were immunoprecipitated to detect ubiquitination. (b) Knockdown of PKM2 decreases p53 ubiquitination. HCT-116 cells were infected with shcont or shPKM2 lentivirus for 24 h, and then treated with MG132 (20 μM) for 6 h. Cells lysates were prepared by using SDS lysis buffer and ubiquitin proteins were immunoprecipitated to detect ubiquitination.

To further identify the possible mechanisms responsible for decreased p53 protein by PKM2 expression, the expression of p53 was detected in PKM2-depletion HeLa, HCT-116 and HL-7702 cells. The results showed that p53 mRNA had no change, but p53 protein level were restrained in PKM2 knockdown cells (Figure 5A, 5B and Supplementary Figure S4B). Moreover, the results clearly indicated that the half-life of p53 was longer in PKM2 knockdown cells (Figure 5C (a) and 5D (a)). And after MG132 treatment, the decreased p53 protein level could be rescued, further indicating that PKM2 depletion increased p53 protein abundance by increasing protein stability (Figure 5C (b) and 5D (b)). Ubiquitination of p53 was also detected and was decreased in PKM2 knockdown cells (Figure 5E). These data confirmed that PKM2 was an important regulator of p53 degradation.

### PKM2 directly binds with both p53 and MDM2, and promotes p53 ubiquitination

**Figure 6:**
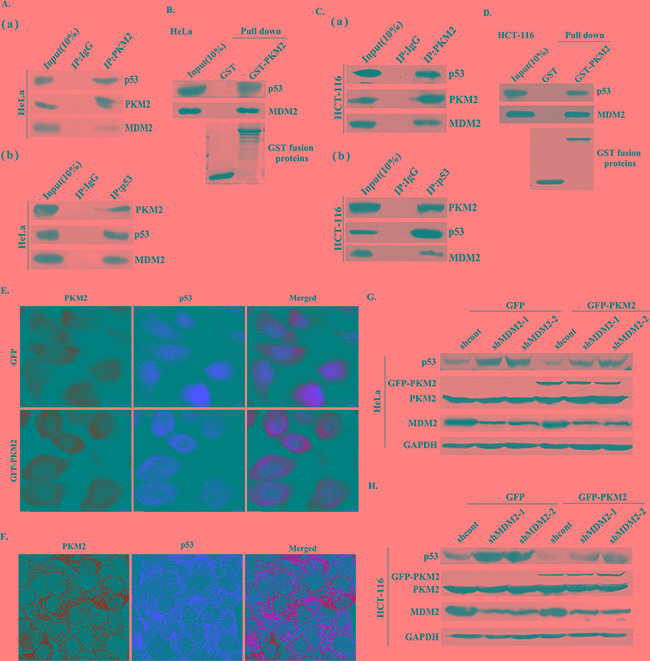
PKM2 binds with both p53 and MDM2 **A.** (a) and (b) Endogenous PKM2 binds to both p53 and MDM2. HeLa cell lysates were used for immunoprecipitation and immunoblotting. **B.** PKM2 directly interacts with p53 and MDM2. HeLa cell lysates was incubated with purified GST or GST-PKM2 protein coupled to GSH-Sepharose. Proteins retained on Sepharose were blotted with the indicated antibodies. **C.** (a) and (b) Endogenous PKM2 binds to both p53 and MDM2. HCT-116 cell lysates were used for immunoprecipitation and immunoblotting. **D.** PKM2 directly interacts with p53 and MDM2. HCT-116 cell lysates was incubated with purified GST or GST-PKM2 protein coupled to GSH-Sepharose. Proteins retained on Sepharose were blotted with the indicated antibodies. **E.** Colocalization of GFP-PKM2 and p53. HeLa cells transfected with GFP or GFP-PKM2 was immunostained with TRITC-conjugated anti-p53 antibody. **F.** Colocalization of endogenous PKM2 and p53. HeLa cells were stained with FITC-conjugated anti-PKM2 and TRITC-conjugated anti-p53 antibodies. **G.** and **H.** MDM2 is mediated the PKM2-induced p53 decrease. HeLa and HCT-116 cells were co-transfected with GFP or GFP-PKM2 and shcont or shMDM2, the expression of p53 was detected by western blot.

To study how PKM2 regulated p53 stability and ubiquitination, we next examined whether PKM2 could interact with p53 and MDM2. MDM2 is an important E3 ligase of p53, and therefore, of vital importance for regulating p53 stability [20]. Interestingly, we found that PKM2 could bind with both p53 and MDM2 (Figure 6A (a) and 6C (a)). Reciprocally, p53 also could bind with PKM2 and MDM2 (Figure 6A (b) and 6C (b)). To test whether the interaction is direct, we purified GST-PKM2 protein and performed an in vitro binding assay. As shown in Figure 6B and 6D, PKM2 could directly pull down p53, as well as MDM2, suggesting a direct interaction between PKM2 and p53, MDM2. The interaction of PKM2 and p53 was further examined by immunostaining assays. Ectopically expressed GFP-PKM2 and p53 were co-localized in the cytoplasm of HeLa cells (Figure 6E). Similarly, endogenous PKM2 was co-localized with endogenous p53 (Figure 6F). We next introduced the shMDM2 into the PKM2 overexpressed cells to determine the involvement of MDM2 in PKM2 regulating p53, the results demonstrated that the decreased expression of p53 was restored following the MDM2 knockdown (Figure 6G and 6H). These data indicated that PKM2 promoted p53 ubiquitination through binding to p53 and MDM2, moreover, MDM2 was mediated the process.

### The dimeric PKM2 plays a dominant role in regulating p53 expression

**Figure 7:**
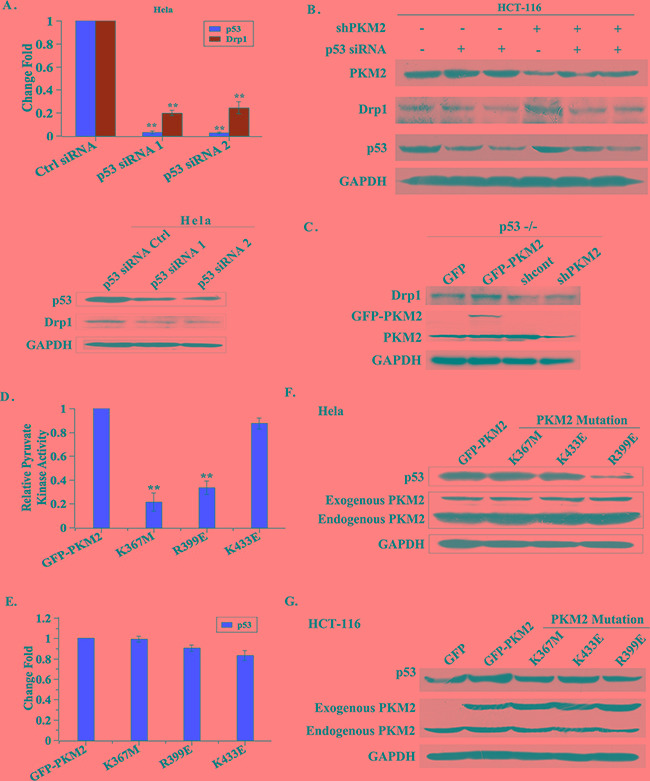
The dimeric PKM2 plays a dominant role in regulating p53 expression **A.** qPCR and western blot evaluation of p53 and Drp1 mRNA and protein expression in HeLa shPKM2 cells in which p53 was knocked down by siRNA. The cells were collected for analysis after 48 h transfection. The cells transfected with non-target siRNA (ctrl siRNA) were as control. *p < 0.05, **p < 0.01. **B.** Western blot analysis of p53 and Drp1 expression in HCT-116 shPKM2 cells in which p53 was knocked down by siRNAs. **C.** Drp1 expression in HCT-116 p53^−/−^ cells. HCT-116 p53^−/−^ cells were infected with GFP, PKM2, shcont or shPKM2 lentivirus for 48 h and cell lysates were analyzed by western blot. **D.** HeLa cells were infected with PKM2, PKM2-K367M, PKM2-R399E or PKM2-K433E lentivirus for 48 h, the pyruvate kinase activity was evaluated by a pyruvate kinase activity assay kit according to the manufacturer's instructions. **p < 0.01. **E.** HeLa cells were infected with PKM2, PKM2-K367M, PKM2-R399E or PKM2-K433E lentivirus. 48 h later, the p53 expression level was evaluated by qPCR. **F.** and **G.** HeLa and HCT-116 cells were infected with PKM2, PKM2-K367M, PKM2-R399E or PKM2-K433E lentivirus. 48 h later, the p53 expression level was evaluated by western blot.

To understand whether the induction of mitochondrial fusion was exclusively mediated by p53, we employed p53 siRNA to knockdown p53 and evaluated Drp1 expression in PKM2-knockdown HeLa, HCT-116 and HL-7702 cells. The results demonstrated that the Drp1 expression was considerably reduced in cells transfected with p53 siRNA for 48 h, compared to cells transfected with control siRNA (Figure 7A, 7B and Supplementary Figure S4C). In addition, whether overexpression of PKM2 or knockdown of PKM2, the expression of Drp1 was not obviously changed in HCT-116 p53^−/−^ cells (Figure 7C). These results indicated that p53 was involved in PKM2-induced mitochondrial abnormality.

Our studies and others’ demonstrate that expect for the pyruvate kinase activity, PKM2 also functions as a protein kinase in tumor cells. The relationship between the p53 expression and PKM2 activity was further studied. We infected HeLa cells with lentivirus mediated PKM2 mutation vectors: K367M, K433E, R399E as described previously [21]. The results of pyruvate kinase activity showed that K367M and R399E mutants had much lower pyruvate kinase activity than that of their wild-type counterpart. The pyruvate kinase activity of the K433E mutant was similar to that of PKM2 overexpressed cells (Figure 7D). Moreover, the result showed that p53 mRNA expression level in these mutants was basically unchanged (Figure 7E). Western blot result showed that p53 protein abundance was equal in K367M and K433E mutant cells compare to PKM2 overexpressed cells, and that was notably decreased in R399E mutant cells (Figure 7F and 7G), in which PKM2 mainly forms dimers. These results suggested that the dimeric PKM2 played a dominant role in regulating p53 expression.

### PKM2 is overexpressed in cervical cancer tissues and its expression is reversely correlated with Drp1 expression in clinical samples

**Figure 8:**
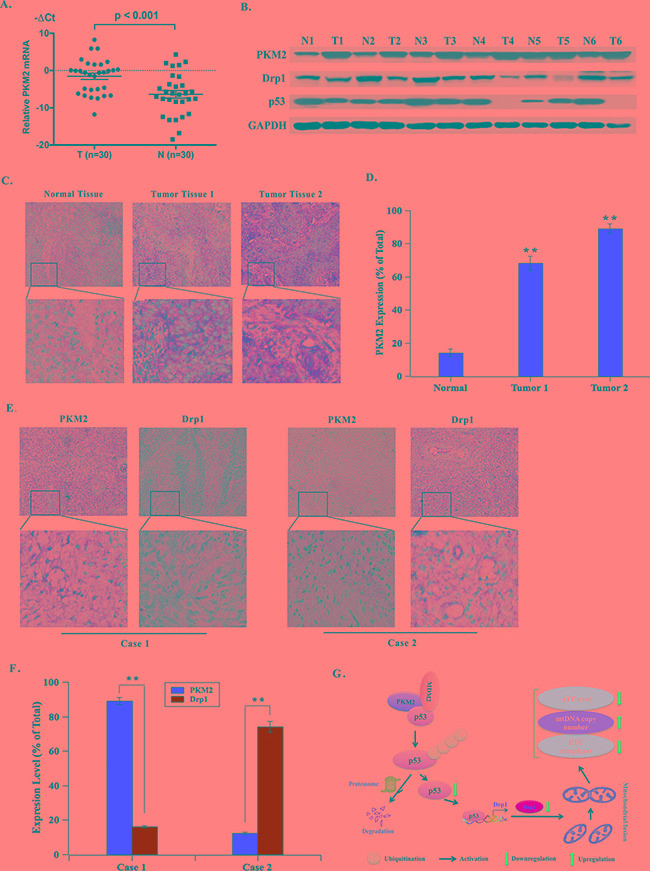
PKM2 is overexpressed in cervical cancer tissues and its expression is reversely correlated with Drp1 expression in clinical samples **A.** qPCR evaluation of PKM2 mRNA expression in 30 pairs of fresh cervical cancer tissues. (T, tumorous tissue; N, nontumorous tissue). **B.** Representative western blot of PKM2, Drp1 and p53 protein expression in 30 pairs of fresh cervical cancer tissues. (T, tumorous tissue; N, nontumorous tissue). **C.** PKM2 expression was determined by IHC. Representative images of strong/weak staining in clinical samples of cervical cancer and negative staining in the nontumorous samples were showed. **D.** The statistical analysis of PKM2 expression. **p < 0.01. **E.** The relationship of PKM2 and Drp1 in clinical samples was determined by IHC. Representative immunohistochemical images of PKM2 and Drp1 were showed. **F.** The statistical analysis of the correlation between PKM2 and Drp1. **p < 0.01. **G.** The schematic of PKM2 regulating mitochondria dysfunction. PKM2 binds to both p53 and MDM2 to form a ternary complex, promoting p53 ubiquitination and degradation, subsequently its downstream target Drp1. Drp1 reduction then results in mitochondria dysfunction.

Furthermore, we detected the expression of PKM2 in cervical cancer clinical samples. Results showed that PKM2 mRNA was significantly overexpressed in tumorous tissues, compared to the corresponding nontumorous tissues (Figure 8A). Consistently, the protein level of PKM2 was markedly increased in cervical cancer fresh tissues. Simultaneously, the tumor tissues exhibited decreased expression of p53 and Drp1 compared with normal tissues (Figure 8B). The immunohistochemical analysis of a large cohort of cervical cancer clinical samples showed that PKM2 expression in cervical cancer tissues was remarkably higher than that in the adjacent normal cervical tissues (Figure 8C and 8D). Having seen that Drp1 expression was altered with PKM2 expression, we further studied the relationship between PKM2 and Drp1 expression in the cervical cancer tissues of clinical samples. Results showed cases with high PKM2 expression in tumor tissues frequently coincides with low Drp1 expression. However, cases with low PKM2 expression were almost with high Drp1 expression, which was identical with the results of cell lines (Figure 8E and 8F).

## DISCUSSION

Cancer cells are very often characterized by an altered cellular metabolism. Since O. Warburg proposed that cancer cells relied on glycolysis rather than on respiratory metabolism even in the presence of oxygen in 1956, several groups had investigated the role of mitochondria in the onset of the Warburg effect. Both in vivo and vitro experiments have demonstrated that the tumor cells have a mitochondrial dysfunction [22]. In the present study, we demonstrated that overexpression of PKM2 showed an increase of elongated mitochondria and decrease of mitochondrial number and further resulted in mitochondrial dysfunction characterized by decreased ATP production, increased mtDNA copy number and decreased expression of ETC complex.

Accumulating evidence suggests that mitochondrial morphology depends on the continual and balanced fission and fusion events that are controlled by a machinery involving large dynamin-related GTPases that exert opposing effects [23]. In mammals, mitochondrial fusion is mediated by mitofusions (Mfn1 and Mfn2, located in the outer mitochondrial membrane) and optic atrophy gene 1 (Opa1, located in the inner membrane). On the other hand, mitochondrial fission is mediated by fission 1 protein (Fis1, located in the outer mitochondrial membrane) and dynamin-related protein 1 (Drp1, which is mostly cytosolic and translocates to the outer mitochondrial membrane during fission) [18, 24, 25]. We hypothesize that enhanced fusion and/or reduced fission cause PKM2-induced elongated mitochondria. In support of this notion, we detected the expression of these proteins. Interestingly, we found that the mitochondrial fission protein Drp1 was decreased in PKM2 overexpressed cells and increased in PKM2 knockdown cells (Figure 2A-2D). At the same time, the mitochondrial fusion protein Mfn2 was increased in PKM2 overexpressed cells and decreased in PKM2 knockdown cells (data not shown). All of this confirmed that PKM2-overexpressing could induce mitochondrial fusion, and then resulted in mitochondrial dysfunction.

The p53 protein stability is principally regulated by ubiquitination of the protein. P53 consists of several different domains: transactivation domain, DNA-binding domain, tetramerization domain, and C-terminal regulatory domain [20]. The Ring domain E3 ubiquitin ligase MDM2 (mouse double minute-2) is the most important p53 regulator and interacts with the DBD region and ubiquitinates p53, promoting its proteasomal degradation [26]. Some proteins compete with MDM2 for binding to p53 transactivation domain and DBD domain and thereby prevent p53 from ubiquitination [27]. In our study, we demonstrated that PKM2 could directly bind with both p53 and MDM2 to form a ternary complex to promote p53 ubiquitination, thereby promoting its degradation (Figure 8G).

Enhanced expression of PKM2 has been observed in various types of cancers and responsible for their poor prognosis, such as gastric cancer [28] and prostate cancer [29]. In this study, we found that in a large cohort of cervical cancer clinical samples, PKM2 expression was markedly increased. The reverse connection of PKM2 and Drp1 was further confirmed in clinical samples. Samples with low PKM2 were frequently accompanied with high Drp1 expression.

Collectively, the findings presented here provide a new perspective for the influence of glycolysis on mitochondrial function, in which high-expression of PKM2 leads to mitochondrial dysfunction through extremely mitochondrial fusion. The identification of potential targets of PKM2 and other possible mechanisms will be helpful to fully understand the oncogenic function of PKM2 and represent a novel therapeutic target for cancer therapy and prevention.

## MATERIALS AND METHODS

### Antibodies and reagents

The RPMI-1640 medium, DMEM-F12 medium and heat-inactivated fetal bovine serum (FBS) were from GIBCO (Gaithersburg, MD, USA). The RNAiso Plus was purchased from Takara (Shiga, Japan). Trans*Script* First-Strand cDNA Synthesis SuperMix and Trans*Start* Top Green qPCR SuperMix were purchased from TransGen (Beijing, China). PKM2 antibody was obtained from Cell Signaling Technology (Danvers, MA, USA). Antibodies for p53, Drp1 and ETC complexes were obtained from Proteintech (Chicago, USA). Antibody against GAPDH was from Bioworld Technology (Minneapolis, MN). Actinomycin D (RNA synthesis inhibitor), Cycloheximide (CHX, protein synthesis inhibitor) and MG-132 (the proteasome inhibitor) were obtained from Sigma (St. Louis, USA).

### Cell culture and transfection

The human cervical cancer cell line HeLa and human normal liver cell line HL-7702 were grown in RPMI 1640 supplemented with 10% FBS and antibiotics. The human colon cancer cell line HCT-116 p53^−/−^ was kindly provided by Prof. Wu Mian (University of Science and Technology of China). HCT-116 and HCT-116 p53^−/−^cells were grown in DMEM-F12 supplemented with 10% fetal bovine serum (FBS) and antibiotics. All cells were cultured in a 37°C, 5% CO_2_ incubator.

Transfection of plasmid or siRNA was performed using Turbofect (Thermo Scientific, MA, USA) or Attractene Transfection Reagent (QIAGEN, Valencia, CA). The sequences of siRNAs against p53 were displayed in supplementary Table S1. For lentiviral packaging, the packaging plasmid psPAX.2, the envelope plasmid pMD2.G and the plvx-AcGFP-PKM2-puro vector or the pLKO.1-shcont or pLKO.1-shPKM2 vector were triple transfected into HEK-293T cells and the supernatant fraction containing lentiviral particles was harvested at 48 and 72 h, respectively, after transfection. HeLa, HL-7702 and HCT-116 cells were infected in the presence of 8 μg /mL polybrene (Millipore, Billerica, MA).

### Observation of mitochondrial morphology

To observe mitochondrial morphology, cells were pre-incubated with 100 nM MitoTracker® Red CMXRos (Invitrogen) for 15 min, fixed in 3.7% paraformaldehyde (PFA) for 15 minutes and permeabilized with ice-cold acetone for 5 min at room temperature. The nucleus was counterstained with DAPI. Mitochondrial morphology was observed using confocal microscopy (Olympus, Japan). To obtain quantitative data, a number of cells (n=100) were typically imaged. Morphological measurements were made using Image J by automated object identification with user-defined thresholds for pixel intensity and size.

### RNA isolation and qRT-PCR

The total RNA isolation and qRT-PCR analysis were performed as previously described [30]. The primer sequences were listed in supplementary Table S2.

### Western blotting analysis and immunoprecipitation

The Western blotting analysis was performed as previously described [30]. For immunoprecipitation, HeLa or HCT-116 cells were harvested and lysates were prepared in NP-40 lysis buffer (50 mM Tris-HCI [pH 7.4], 0.5% NP-40, 150 mM NaCl) with protease inhibitor cocktail (Roche Diagnostics, Quebec, Canada). Cell lysates were incubated with 2 μg antibody overnight at 4 °C. Then 40 μL protein A/G-agarose (Beyotime) was added and rotated continuously at 4 °C for 3 h. The beads were washed three times with a PBS buffer. Immunoprecipitates were suspended in 2xSDS sample buffer and boiled at 100°C for 5 min, and then subjected to SDS-PAGE and Western blotting.

### Detection of cellular ATP levels

Cellular ATP levels were measured using an ATP Assay Kit (Beyotime, China) according to the manufacturer's instructions. Briefly, 24 h after transfection, cells were harvested and lysised, then centrifuged at 12,000 g for 5 min. In 6-well plates, 200 μL supernatant was mixed with 100 μL ATP detection working dilution. The luminance (RLU) was measured by a fluorescence microplate reader (Thermo Scientific Varioskan Flash, USA). Standard curve was also generated and the protein concentration was determined by the BCA assay (Thermo Scientific). Total ATP levels were expressed as nmol/mg protein. The relative ATP level was calculated according to the following formula: relative ATP level = ATP value / protein concentration.

### Quantification of mtDNA copy number

Genomic DNA was extracted from HeLa and HCT-116 cells using QIAamp DNA Mini kits (QIAGEN, Valencia, CA) according to manufacturer's instructions. Relative mtDNA copy number was measured by a quantitative real-time polymerase chain reaction (PCR)-based method as previously described [31].

### P53 ubiquitination assay

PKM2 overexpressing or knockdown HeLa and HCT-116 cells were treated with 20 mM MG132 for 4 h, then cells were lysed and immunoprecipitated with p53 or ubiquitin antibody. The immunocomplexes were analyzed by western blot.

### Immunofluorescence staining

Cells were seeded on coverslips and washed twice with cold PBS, then fixed with 4% paraformaldehyde, and permeabilized with 0.1% Triton X-100 for 15 min. Cells were blocked with 5% BSA and incubated with antibodies as indicated, followed by a TRITC-conjugated anti-rabbit IgG and a FITC-conjugated anti-mouse IgG antibody. The cells were mounted with DAPI and the images were acquired with an Olympus microscope.

### GST pulldown assay

GST and GST-PKM2 fusion proteins were purified in a standard way and incubated with cell lysates. After three washes, the bound proteins were separated with SDS-PAGE and blotted with indicated antibodies.

### Tissue specimens and immunohistochemistry

A total of 30 pairs of fresh, surgically resected specimens (30 cases of tumorous tissues and 30 cases of nontumorous tissues) and 64 cases of clinical sample sections (40 cases of tumorous tissues and 24 cases of nontumorous tissues) were collected from the First Hospital Affiliated with Shanxi Medical University and the Armed Police Hospital (Shanxi, China). All sections were stained with antihuman PKM2 and Drp1. Staining was performed using a universally labeled streptavidin−biotin kit according to the standard protocol. The percentage of cells was calculated by Image Pro-Plus 6.0 software.

### Statistical analysis

All results were depicted as mean values ± standard deviations (SDs). The statistical software SPSS16.0 was used in data processing and analyzing. Statistical analysis of the differences was analyzed using Student's *t*-test, and *P* < 0.05 was considered as significant.

## SUPPLEMENTARY MATERIALS FIGURES AND TABLES


