# Supplementary material for: Overexpression of PKM2 promotes mitochondrial fusion through attenuated p53 stability
Source: Oncotarget. 2016 Oct 27;7(47):78069–82. doi: 10.18632/oncotarget.12942 (PMC5363644; doi:10.18632/oncotarget.12942)
Supplement: Supplementary file 1 [file oncotarget-07-78069-s001.pdf]

Overexpression of PKM2 promotes mitochondrial fusion through attenuated p53 stability

SUPPLEMENTARY FIGURES AND TABLES

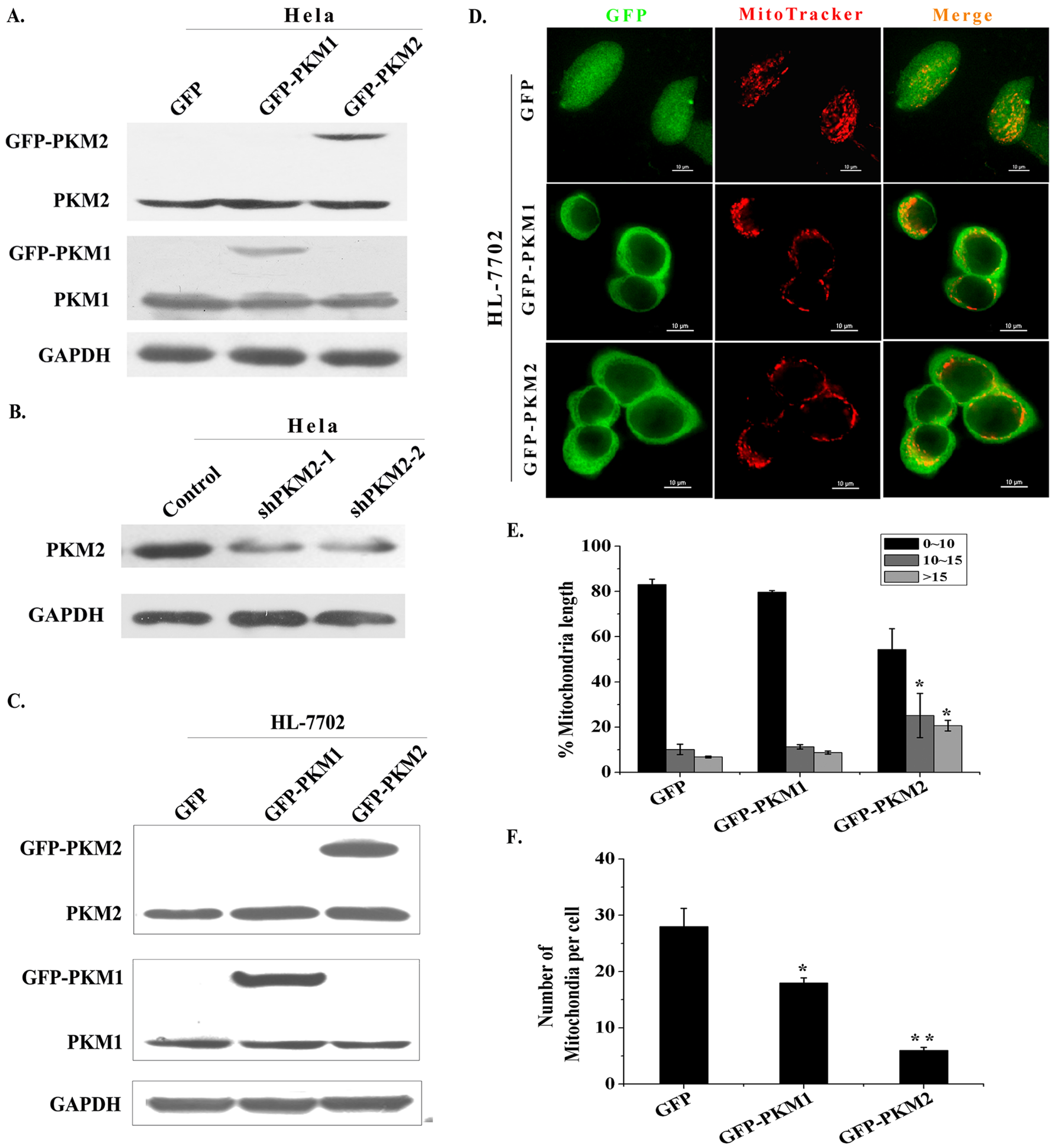

Supplementary Figure S1: PKM2 overexpression induced abnormal mitochondrial morphology. A. The protein expression level of PKM1 and PKM2 in PKM1 or PKM2 overexpressed HeLa cells. **B.** The protein expression level of PKM2 in PKM2-knockdown HeLa cells. **C.** The protein expression level of PKM1 and PKM2 in PKM1 or PKM2 overexpressed HL-7702 cells. **D.** Representative images of mitochondrial morphology. HL-7702 cells were infected with lentivirus expressing GFP, PKM1or PKM2 for 24 h. Mitochondrial morphology was observed by confocal fluorescent microscope with Mitotracker dye (Green: GFP, Red: Mitotracker dye). Scale bar =10 µm. **E.** Statistical results of mitochondrial morphology. After transfection 24 h, over 100 transfected cells were categorized into one of three groups depending on mitochondrial morphology. *p < 0.05. **F.** Quantitative analysis of mitochondrial number. *p < 0.05, **p < 0.01.

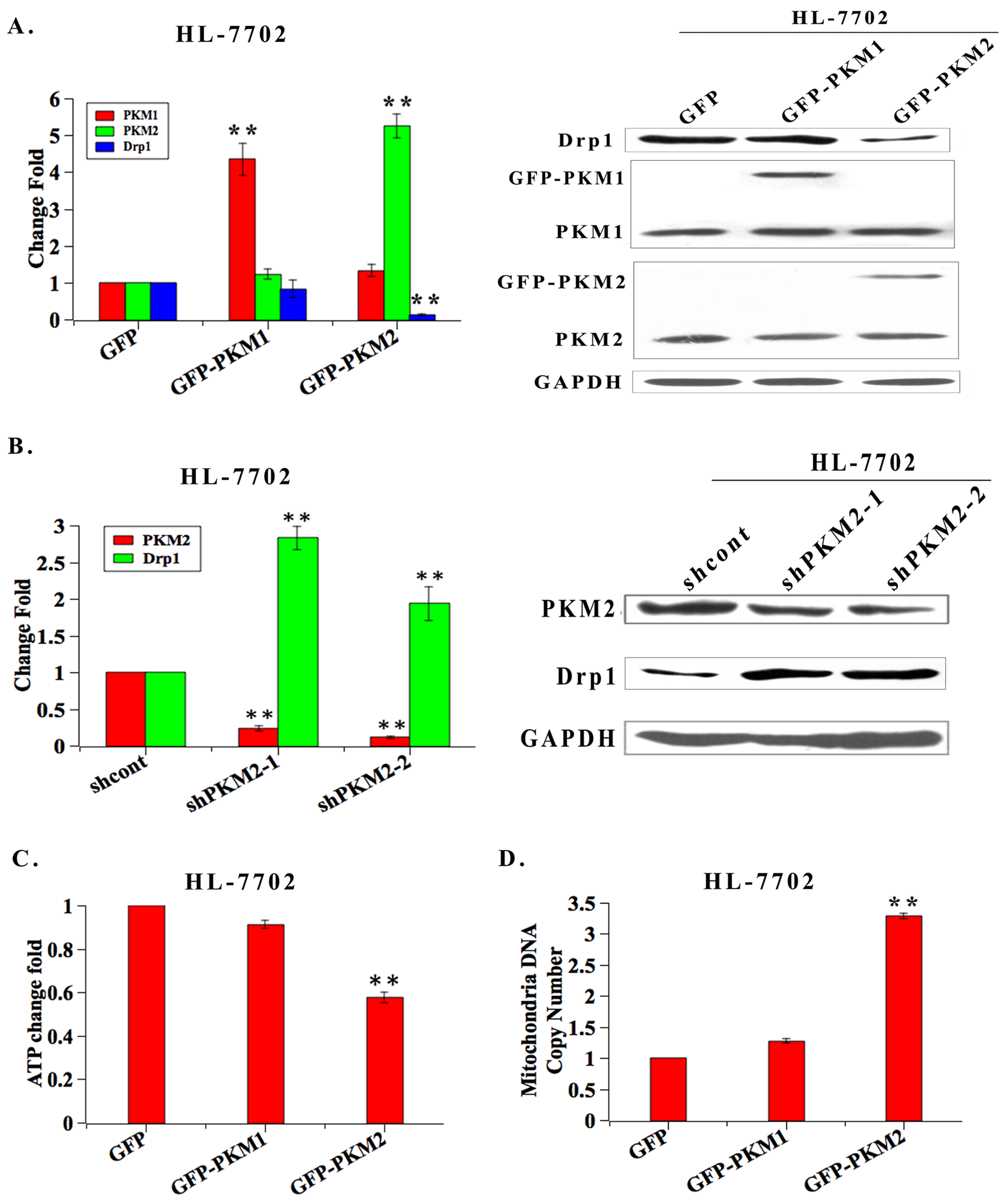

Supplementary Figure S2: PKM2 induces abnormal mitochondrial fission / fusion events and mitochondrial dysfunction. **A.** The mRNA and protein expression levels of Drp1 are decreased in PKM2 overexpressed cells. HL-7702 cells were infected with GFP, PKM1or PKM2 lentivirus for 24 h and Drp1 expression was evaluated by qPCR and western blot. **p < 0.01. **B.** The mRNA and protein expression levels of Drp1 are increased in PKM2 knochdown cells. HL-7702 cells were infected with shcont, shPKM2-1or shPKM2-2 lentivirus for 24 h and Drp1 expression was evaluated by qPCR and western blot. **p < 0.01. **C.** PKM2 overexpression decreases the ATP production. Intracellular ATP was determined using a luciferase-based luminescence assay kit according to the manufacturer's instructions. **p < 0.01. **D.** Mitochondrial DNA copy number is increased in PKM2 overexpressed cells. Total DNA was extracted, and relative levels of mtDNA copy number (ND1) were determined by qPCR. The nuclear gene HGB was used for normalization. **p < 0.01.

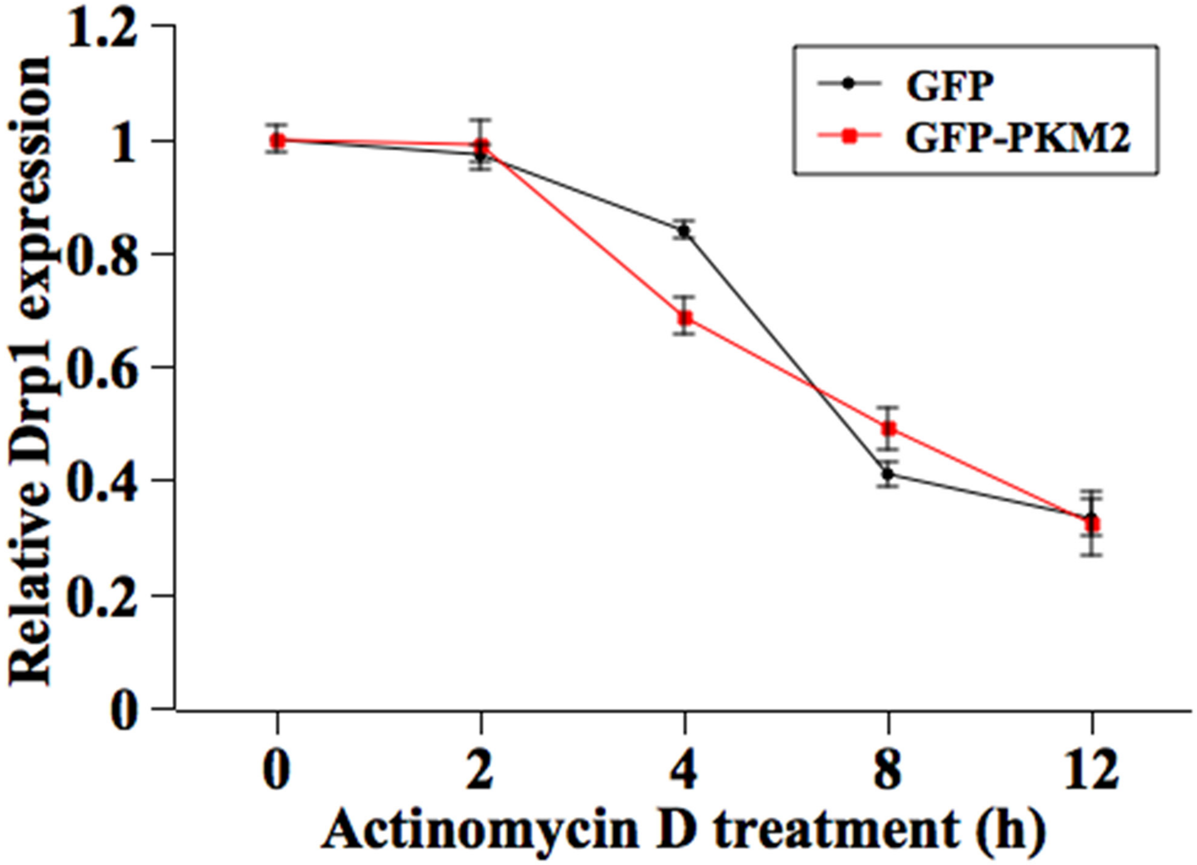

Supplementary Figure S3: PKM2 modulates Drp1 mRNA at the transcriptional level. HeLa cells were infected with lentivirus expressing GFP or PKM2 for 24 h, and then exposed to 5 µg/ml actinomycin D for 2 h, 4 h, 8 h, 12 h. The quantitative assessment of Drp1 mRNA levels showed that PKM2 had no effects on the stability of the Drp1 mRNA.

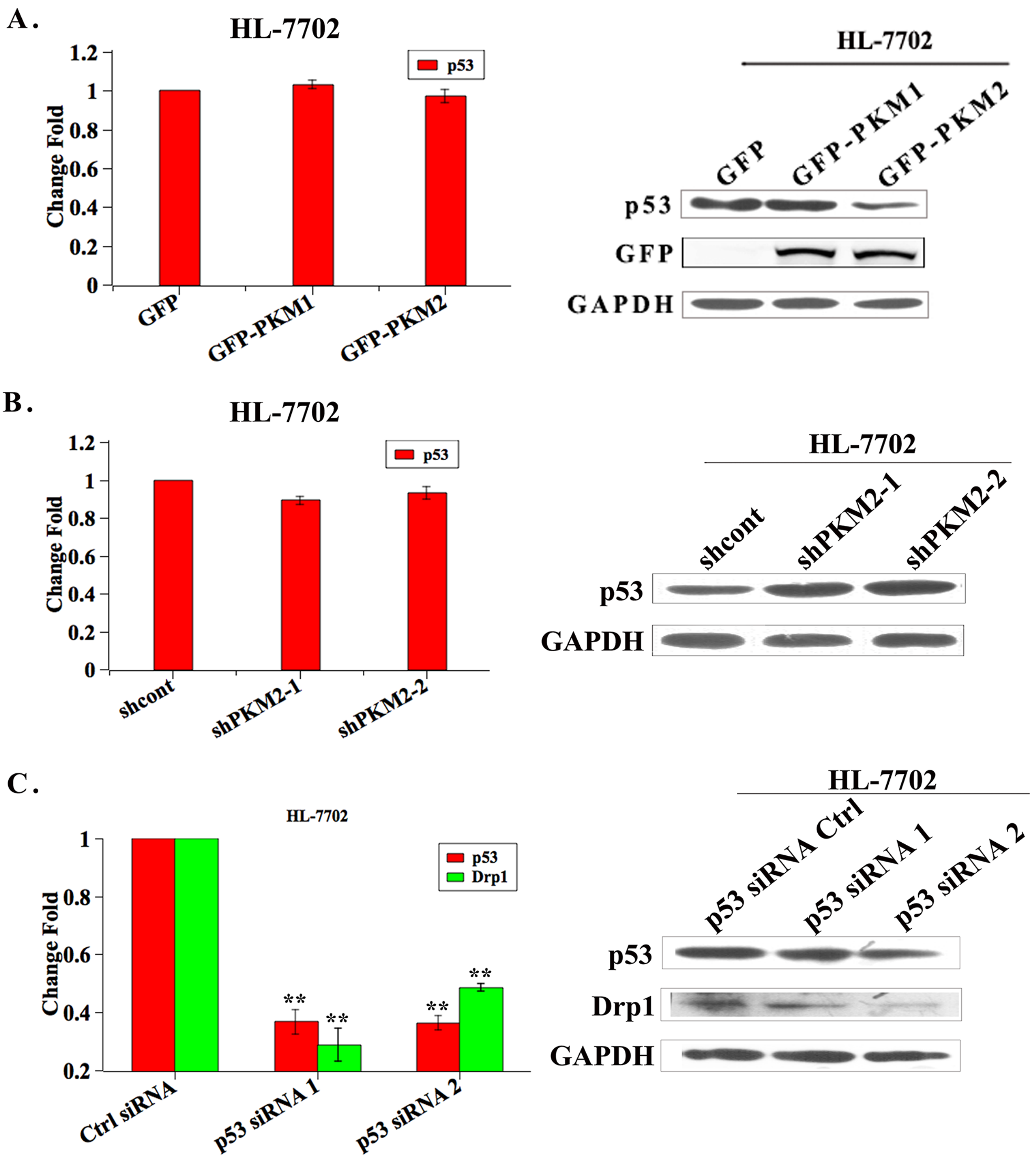

Supplementary Figure S4: P53 is involved in PKM2-induced mitochondrial fusion. A. The p53 protein expression level is decreased in PKM2 overexpressed cells. HL-7702 cells were infected with GFP, PKM1or PKM2 lentivirus for 24 h and p53 mRNA and protein expression level were evaluated by qPCR and western blot. **B.** The p53 protein expression level is increased in PKM2 knockdown cells. HL-7702 cells were infected with shcont, shPKM2-1or shPKM2-2 lentivirus for 24 h and p53 mRNA and protein expression level were evaluated by qPCR and western blot. **C.** qPCR and western blot evaluation of p53 and Drp1 mRNA and protein expression in HL-7702 shPKM2 cells in which p53 was knocked down by siRNA. The cells were collected for analysis after 48 h transfection. The cells transfected with non-target siRNA (ctrl siRNA) were as control. **$p < 0.01$.

Supplementary Table S1: The sequences of siRNAs against p53

Gene	siRNA Sequence (5’-3’)
siRNA-1	UGGUUCACUGAAGACCCAGTT
siRNA-2	CCACCAUCCACUACAACUATT
Control	UUCUCCGAACGUGUCACGUTT

Supplementary Table S2: Primers used for real-time qPCR

Gene	Forward primer (5’-3’)	Reverse primer (5’-3’)
PKM1	GAAGAACTTGTGCGAGCCT	CGTC AGAACTATCAAAGCTGC
PKM2	GCTGCCATCTACCACTTGC	CCAGACTTGGTGAGGACGATT
Drp1	GGTGGGGTTGGAGATGGTGTT	CGCTGTTCCCGAGCAGATAGTT
p53	TCAACAAGATGTTTTGCCAACTG	ATGTGCTGTGACTGCTTGTAGATG
GAPDH	GCACCGTCAAGGCTGAGAAC	TGGTGAAGAACGCCAGTGGA
